# Teaching Psychology Research Methodology Across the Curriculum to Promote Undergraduate Publication: An Eight-Course Structure and Two Helpful Practices

**DOI:** 10.3389/fpsyg.2018.02295

**Published:** 2018-11-27

**Authors:** Stuart McKelvie, Lionel Gilbert Standing

**Affiliations:** Department of Psychology, Bishop's University, Sherbrooke, QC, Canada

**Keywords:** teaching, research methods, course structure, critical discussion, replication

## Goals for teaching research methods to undergraduates

Teaching research methods is challenging because we not only wish to convey formal knowledge and encourage critical thinking, as with any course, but also to enable our students to dream up meaningful research projects, translate them into logical steps, conduct the research in a professional manner, analyze the data, and write a report in APA style. We also wish to spark interest in research, but in teaching undergraduates we have learned how elusive these goals can be (McKelvie, 1994). Even faculty have not mastered research design and writing. From serving as journal reviewers, we have found that many submissions show flaws such as elementary errors of logic (e.g., using a null control condition instead of a placebo treatment), tangled statistics, missing graphs, and ungrammatical, unclear writing that violates APA rules. Yet these manuscripts are sometimes written by people with doctorates and years of experience. Moreover, published papers may contain egregious faults (Standing and McKelvie, 1987). And although we have both published widely, we still hone our skills. It requires optimism to expect that a typical undergraduate will do better, after just a year or two of studies in psychology. In this paper, we describe a *systematic set of methodology courses* and two *specific practices* that we think can help.

## Methodology courses as the backbone of our psychology program

How can methodology courses promote undergraduate involvement in publishable research? In our undergraduate liberal arts institution in Québec, where the Bachelor's degree normally takes 3 years following 2 years of college, we require more, rather than less: our psychology program has evolved since the 1960s to require a solid backbone of mandatory methods-related courses that is considerably more extensive than in most universities (McKelvie, 2000). Psychology majors take two consecutive introductory statistics courses in the first academic year, reaching the level of two-way ANOVA. Simultaneous with the second course, they take an introductory research methods course with lectures and discussion of important concepts, including theory, and involvement (participation, testing, writing) in instructor-planned projects that usually extend past research. In the second year, intending honors students take an advanced methods course that builds on the first one. It uses the same text, and continues active participation in project work. An unusual requirement (McKelvie, 2000) is a course in Psychometrics and Psychological Testing, reflecting our belief in the importance of measurement. In the third year, students with a program average of 80% or better, *and* a combined average of 75% in the advanced research methods course plus the second statistics course, may enter the honors program. They take a multivariate statistics course, and produce an idea for a data-based honors thesis under the direction of a main and a secondary supervisor. Students are encouraged to create their own research question on a topic of their choosing. Over two semesters, they discuss this project in a seminar course and write a formal proposal, and then in the thesis course, they conduct the research and write the report.

These eight required methods-related courses produce well-grounded and motivated honors graduates, and gives them an opportunity to publish. Our students accept with good grace the challenge of this program, where only about one-fifth of them will obtain an honors degree rather than a major, and our departmental numbers have risen considerably over the years.

## Traditional solutions for teaching students to grapple with research methods

A traditional solution in teaching research methods and exposing students to the research process has been first to lead them through a series of short, pre-packaged lab projects that demonstrate some well-established phenomena, and require brief write-ups, likely in APA format. This approach still has merit at the introductory stage, and the Online Psychology Laboratory experiments provided by the APA (https://opl.apa.org/) are valuable exercises for the neophyte. However, it appears that today more emphasis is commonly placed on original individual methods projects, perhaps to prepare students for an honors thesis, where they commonly choose the topic.

The basic problem is that too many original student course projects lack a valid idea to test, coherent methods, and a valid formal design. Additionally, sample sizes are usually too low, yielding inadequate statistical power. More fundamentally, students habitually gravitate to correlational relationships rather than to randomized controlled experiments. They must learn to better justify proposals for non-experimental research.

## Two specific research methods teaching strategies

### Class discussion of published articles (introductory methods course)

Critical class discussion of published articles can spark student interest in research papers and help them design better studies (McKelvie, 1994, 2013). Papers are carefully chosen to capture student attention and expose them to methodological issues. Study questions focus on important points in each reading. Students are also encouraged to generate their own critical comments and queries (McKelvie, 2013). This approach sits well with the typical case-oriented contents of leading methods texts (e.g., Morling, 2018).

Five study questions are common to all papers (McKelvie, 2013): What type(s) of research method is (are) involved? In particular, is it a true experiment? What inferential statistics were used? Were they appropriate? What is (are) the sources of the problem (theory, past research, practical intervention, everyday life)? One example is Motley and Camden's (1985) study of sexual double entendres in lexical selection. It employed both experimental (manipulation of experimenter attractiveness) and non-experimental methods (sexual anxiety as a subject variable). Independent samples *t*-tests, chi square, and ANOVA were used. The study was based on theory and on everyday life. Another example is Milgram's (1963) seminal observational study of obedience. It is non-experimental, only contains descriptive statistics, and is based on everyday life. Students find it challenging to identify the research method. Realizing that “laboratory” does not mean “experiment” is a valuable lesson. A complete list of study questions and discussion papers is available (see link to McKelvie, 2013).

### Replication projects (advanced methods course)

Although the traditional laboratory approach has merit, the projects may only be demonstrations, or suffer from inadequate sample size. Original individual student projects share this problem, and have other difficulties.

One solution is for all the class to work on the *same* research project, created by the instructor in an area of their expertise. This study may be *original* as described in detail by LoSchiavo (2019). Advantages are that sample sizes will be healthy, increasing the likelihood of publication, and that students are motivated to create new knowledge. Alternatively, the instructor can plan a *replication* study, selecting a paper from the literature that is widely quoted and of manageable scope, and leading the class through either a conceptual or (better) an exact replication of the target study. Sample sizes will again be healthy, but other advantages are that planning is simplified, a rationale for the study exists, the method is pre-established, and students are educated about the replication debate (Maxwell et al., 2015). Their involvement may also be promoted by the realization that we can grapple together with the same issues as published authors, and that they are engaging in “real” research that is potentially publishable. The experience also prepares honors students in the seminar and thesis courses to create an original project, or one that replicates and extends previous work (e.g., Benmergui et al., 2017).

#### The parallel teams approach

A class may be divided into teams which each work on a different target article. Preferably, the teams can each try to replicate the *same* paper, which has the advantage of maximizing *N*, and we note that replications to be adequately powered should use *more* than the number of participants listed in the target article (Simonsohn, 2015). Additionally, the team results can be compared for consistency, before pooling the data to increase power. When the teams are of perhaps half a dozen members each, they can function most effectively as small, cohesive groups under the direction of an elected or designated team leader (for practical details see Standing, 2016).

An example of a project using four parallel replication teams is described by Standing et al. (2016). This study successfully replicated Experiment 8 in the study by Gailliot et al. (2007), which made the controversial claim that self-control can be raised by consuming glucose rather than a placebo drink. As possible authorship is motivating to many students, the four team leaders here were included as coauthors of the instructor, with the remaining members of the class acknowledged in a footnote. Alternatively, all members of a class team may be listed as authors, as with a previous attempt focusing on the claim that priming a participant with a trait such as “intelligence,” or a stereotype such as “professor,” raises their cognitive performance in the form of Trivial Pursuit scores (Dijksterhuis and van Knippenberg, 1998). This replication attempt did not succeed (Roberts et al., 2013). Subsequently both a 9-experiment study (Shanks et al., 2013), and a preregistered replication study, involving 40 labs, have likewise failed to replicate the target study's results (O'Donnell et al., 2018).

The results of student replication projects are most effectively communicated by posting them as summaries on the PsychFileDrawer.org website, which provides a refereed “Archive of Brief Reports of Replication Attempts in Experimental Psychology.”

## Challenges

Major challenges in teaching research methods include students' limited ability to (a) build a study that makes a clear prediction with rival hypotheses, and (b) to think clearly and logically through key issues such as randomization, control conditions, double-blind testing, counterbalancing, power, sample size, experimenter effects, and demand characteristics (McKelvie, 1994). Another challenge is to have the student exert tighter controls in non-experimental studies [e.g., match groups on subject variables (Lemieux et al., 2002), and include a dependent variable on which no difference is expected]. Fundamentally, we ask honors students to propose a new study that is valid and interesting, with a clear connection to previous work. We confront these issues explicitly, in class and in personal interactions with students. Another major issue is that of obtaining prior approval from institutional research ethics boards, which requires careful time scheduling and attention to detail in the required documentation. To plan ahead of time, pilot testing is vitally needed, and this testing itself may require ethics approval, leading possibly to an infinite logical regress unless common sense is applied.

The problems of writing skills, correct citation, and APA format are also pervasive. We find it useful to break reports into sections (staggered over time), to allow resubmission (after editing by the instructor), and to encourage students to consciously imitate the style of the APA model manuscript (American Psychological Association., 2010, pp. 41–53), rather than to memorize formal rules. Students also receive detailed handouts explaining these rules (https://www.ubishops.ca/wp-content/uploads/McKelvie_GuidetowritingreportsAPA6thedition-converted.pdf; http://www.ubishops.ca/wp-content/uploads/plagia04.pdf).

## Outcomes

In addition to the replications published *in PsychFileDrawer*, the present approach, developed over several decades, has yielded a variety of PsycINFO-listed refereed articles with undergraduates: 71 and 50 papers for the present authors, respectively. Most papers are based on honors theses (e.g., McKelvie and Demers, 1979; Knight and McKelvie, 1986; Martel et al., 1987; Shackell and Standing, 2007; Standing et al., 2008, 2014; Sigal and McKelvie, 2012; Clohecy et al., 2015; Morin-Lessard and McKelvie, 2017), where students usually earn the right to primary authorship because they proposed and conducted the research, even if we led the conversion from thesis report to manuscript. In other cases (e.g., McKelvie et al., 2013), where students assisted in our projects, we were primary authors. Disputes can be avoided by clarifying authorship ground rules at the outset.

As an example (Benmergui et al., 2017), one investigation attempted to replicate a report (Beauchamp, 2002) that false recall in the Deese-Roediger-McDermott-Read-Solso (DRMRS) procedure would be smaller when the materials were pictures rather than words. Items for remembering are constructed around a theme that is not on the list (e.g., thread, pin, sewing around needle). False memory occurs when the theme word (needle) is recalled. This replication was successful, and the experiment extended previous research with new materials, new conditions, and a measure of confidence.

## Conclusion

Involving undergraduates in the publication process is not easy, but we believe that the present seeming success is related in part to our cumulative course structure, to the explicit identification and discussion of challenges, and to the two systematic exercises outlined here.

## Author contributions

Both authors listed have made a substantial, direct and intellectual contribution to the work, and approved it for publication.

### Conflict of interest statement

The authors declare that the research was conducted in the absence of any commercial or financial relationships that could be construed as a potential conflict of interest.

## References

[B1] American Psychological Association (2010). Publication Manual of the American Psychological Association, 6th Edn. Washington, DC: American Psychological Association.

[B2] Beauchamp H. M. (2002). Aural, visual, and pictorial stimulus formats in false recall. Psychol. Rep. 91 (3 Pt 1), 941–951. 10.2466/PR0.91.7.941-951 12530749

[B3] Benmergui S. R. McKelvie S. J. Standing L. G. (2017). Beneficial effect of pictures on false memory in the DRMRS procedure. Curr. Psychol. 36, 136–146. 10.1007/s12144-015-9394-y

[B4] Clohecy E. D. Standing L. G. McKelvie S. J. (2015). What enables self-control? A test of glucose, fructose, and vagus nerve activation as possible factors. Psychol. Writings 8, 26–31. 10.5231/psy.writ.2015.1512

[B5] Dijksterhuis A. van Knippenberg A. (1998). The relation between perception and behavior, or how to win a game of Trivial Pursuit. J. Personality Soc. Psychol. 74, 865–877. 10.1037/0022-3514.74.4.865 9569649

[B6] Gailliot M. T. Baumeister R. F. DeWall C. N. Maner J. K. Plant E. A. Tice D. M.. (2007). Self-control relies on glucose as a limited energy source: willpower is more than a metaphor. J. Pers. Soc. Psychol. 92, 325–336. 10.1037/0022-3514.92.2.325 17279852

[B7] Knight L. J. McKelvie S. J. (1986). Effects of attendance, note-taking, and review on memory for a lecture: encoding vs. external storage functions of notes. Can. J. Behav. Sci. 18, 52–61. 10.1037/h0079957

[B8] Lemieux P. McKelvie S. J. Stout D. (2002). Self-reported hostile aggression in contact athletes, no contact athletes and non-athletes. Athletic Insight 4, 42–56.

[B9] LoSchiavo F. M. (2019). Incorporating a professional-grade all-class project into a research methods course. Front. Psychol. 9:e02143. 10.3389/fpsyg.2018.02143 30459692 PMC6232375

[B10] Martel J. McKelvie S. J. Standing L. (1987). Validity of an intuitive personality scale: personal responsibility as a predictor of academic achievement. Edu. Psychol. Measurement 47, 1153–1163. 10.1177/0013164487474033

[B11] Maxwell S. E. Lau M. Y. Howard G. S. (2015). Is psychology suffering from a replication crisis? What does “Failure to teplicate” really mean? Am. Psychol. 70, 487–498. 10.1037/a0039400 26348332

[B12] McKelvie S. J Juillet D. R. Longtin J. V. (2013). Comparing the perceived size of 9 with 221 and 2143: biasing effects of inferred context in a between subjects design. *J. Sci. Psychol* 25–44 Available online at http://psyencelab.com/uploads/5/4/6/5/54658091/perceived_size.pdf

[B13] McKelvie S. J. (1994). Grappling with confusion: student difficulties with research methods. Psychol. Teach. Rev. 3, 125–129.

[B14] McKelvie S. J. (2000). Psychological testing in the undergraduate curriculum. Can. Psychol. 41, 141–148. 10.1037/h0086863

[B15] McKelvie S. J. (2013). Understanding statistics and research methods via discussion of published articles, in Essays from E-xcellence in Teaching, Vol. XII, Chapter 3, eds Holmes J. Baker S. C. Stowell J. R., 16–24. Available online at: http://teachpsych.org/Resources/Documents/ebooks/eit2012.pdf

[B16] McKelvie S. J. Demers E. G. (1979). Individual differences in reported visual imagery and memory performance. Br. J. Psychol. 70, 51–57. 10.1111/j.2044-8295.1979.tb02142.x 486866

[B17] Milgram S. (1963). Behavioral study of obedience. J. Abnormal Soc. Psychol. 67, 371–378. 10.1037/h0040525 14049516

[B18] Morin-Lessard E. McKelvie S. J. (2017). Does writeing rite matter? Effects of surface textual errors on personality trait attributions. *Curr. Psychol*. Available online at: https://link-springer-com.proxy.ubishops.ca:2443/content/pdf/10.1007%2Fs12144-017-9582-z.pdf. (Accessed March 25, 2017).

[B19] Morling B. (2018). Research Methods in Psychology, 3rd Edn. New York, NY: Norton.

[B20] Motley M. T. Camden C. T. (1985). Nonlinguistic influences on lexical selection: evidence from double entendres. Commun. Monogr. 52, 124–135. 10.1080/03637758509376100

[B21] O'Donnell M. Nelson L. D. Ackermann E. Aczel B. Akhtar A. Aldrovandi S.. (2018). Registered replication report: Dijksterhuis and van Knippenberg (1998). Perspect. Psychol. Sci. 13, 268–294. 10.1177/1745691618755704 29463182

[B22] Roberts M. S. Crooks W. Kolody T. J. Pavlovic T. Rombola K. J. Standing L. G. (2013). No Effect on Intelligence from Priming [Replication of Dijksterhuis & van Knippenberg]. Available online at: http://www/PsychFileDrawer.org/chart.phptarget_article=33

[B23] Shackell E. M. Standing L. G. (2007). Mind over matter: mental training increases physical strength. N. Am. J. Psychol. 9, 189–200.

[B24] Shanks D. R. Newell B. R. Lee E. H. Balakrishnan D. Ekelund L. Cenac Z.. (2013). Priming intelligent behavior: an elusive phenomenon. PLoS ONE 8:e56515. 10.1371/journal.pone.0056515 23637732 PMC3634790

[B25] Sigal M. McKelvie S. J. (2012). Is exposure to visual media related to cognitive ability? Testing Neisser's hypothesis of the Flynn effect. J. Articles Support Null Hypothesis 9, 23–49.

[B26] Simonsohn U. (2015). Small telescopes: detectability and the evaluation of replication results. Psychol. Sci. 26, 559–569. 10.1177/0956797614567341 25800521

[B27] Standing L. G. (2016). How to use replication team projects in a research methods course, in Essays from E-xcellence in Teaching, Vol. XV, Chapter 7, eds Altman W. S. Stein L. Westfall J. E., 26–31. Available online at: http://teachpsych.org/Resources/Documents/ebooks/eit2015.pdf

[B28] Standing L. G. Aikins S. Madigan B. Nohl W. (2014). Exceptional achievement and early parental loss: the Phaeton effect in American writers, presidents, and eminent individuals. J. Psychohist. 42, 188–199. 25630195

[B29] Standing L. G. Astrologo L. Benbow F. F. Cyr-Gauthier C. S. Williams C. A. (2016). A successful test of parallel replication teams in teaching research methods. Psychol. Teach. Rev. 22, 49–57.

[B30] Standing L. G. McKelvie S. J. (1987). Psychology journals: a case for treatment. Bull. Br. Psychol. Soc. 39, 445–450.

[B31] Standing L. G. Verpaelst C. C. Ulmer B. K. (2008). A demonstration of nonlinear demand characteristics in the “Mozart effect” experimental paradigm. N. Am. J. Psychol. 10, 553–556.

